# Minimal Cardinality Diagnosis in Problems with Multiple Observations

**DOI:** 10.3390/diagnostics11050780

**Published:** 2021-04-26

**Authors:** Meir Kalech, Roni Stern, Ester Lazebnik

**Affiliations:** 1 Software and Information Systems Engineering, Ben Gurion University of the Negev, Beer-Sheva 8410501, Israel; roni.stern@gmail.com (R.S.); esterla@post.bgu.ac.il (E.L.); 2 Palo Alto Research Center, Palo Alto, CA 94304, USA

**Keywords:** model-based diagnosis, intermittent faults, multiple observations, behavior modes

## Abstract

Model-Based Diagnosis (MBD) is a well-known approach to diagnosis in medical domains. In this approach, the behavior of a system is modeled and used to identify faulty components, i.e., once a symptom of abnormal behavior is observed, an inference algorithm is run on the system model and returns possible explanations. Such explanations are referred to as diagnoses. A diagnosis is an assumption about which set of components are faulty and have caused the abnormal behavior. In this work, we focus on the case where multiple observations are available to the diagnoser, collected at different times, such that some of these observations exhibit symptoms of abnormal behavior. MBD with multiple observations is challenging because some components may fail intermittently, i.e., behave abnormally in one observation and behave normally in another, while other components may fail all the time (non-intermittently). Inspired by recent success in solving classical diagnosis problems using Boolean satisfiability (SAT) solvers, we describe two SAT-based approaches to solve this MBD with multiple observations problem. The first approach compiles the problem to a single SAT formula, and the second approach solves each observation independently and then merges them together. We compare these two approaches experimentally on a standard diagnosis benchmark and analyze their pros and cons.

## 1. Introduction

A diagnosis problem arises when a system does not behave as expected. The goal of diagnosis algorithms is to find the set of faulty components that caused the unexpected behavior of the system. The creation of intelligent medical diagnostic systems has been one of the most profitable fields since the early days of artificial intelligence. Model-Based Diagnosis (MBD) [1] is well-established approach for building diagnostic systems, which have been applied to medical domains [2,3] as well as high-speed trains [4] and other domains [5]. In MBD, a formal model of the diagnosed system is assumed to exist specifying the expected behavior of the system. This model, along with an observation of the system, i.e., a series of inputs to the system and the respected outputs, is then used to deduce candidate diagnoses.

Considering only a single observation of the diagnosed system limits the effectiveness of the diagnosis process since it may not contain sufficient information about the condition of the system. Furthermore, in many real-life systems getting additional observations is feasible and not costly. In these cases, one would like to collect additional observations and take consider them in the diagnostic process, as they may provide extra information on the current problem, and result in a more accurate diagnosis. Indeed, many real-world systems collect multiple observations of the system behavior over time.

In this work, we study the MBD problem when multiple observations of the system, taken in different time steps, are given. We call this the *multi-observations MBD* (MO-MBD) problem. MO-MBD poses additional challenges over the classical diagnosis problem (in which a single observation is given). First, MO-MBD has a higher complexity, as it needs to reason about more information. Second, a faulty component may behave normally in some observations, thus making it more difficult to find. Such faults, which manifest only in some observations, are called *intermittent faults*.

We focus on a model-based approach for solving MO-MBD, i.e., we assume that a model of the system’s behavior is given and infer diagnoses from the model and the observations. There are two types of models in the model-based diagnosis field: weak-fault model (WFM) and strong-fault model (SFM). A system model is said to have a weak-fault model if it only specifies the normal behavior of its components. By contrast, system model with a strong-fault model contains information about the abnormal behavior of its components. For example, a pipe in a system with weak-fault model can be either healthy or faulty, i.e., one can learn from the model about the normal behavior of the pipe, but there is no information about its abnormal functioning. On the other hand, a pipe in a corresponding system with a strong-fault model may have the following behavior modes: healthy, leaked or blocked. Given the behavior mode of a pipe in such a system and the observed input, one can infer the output value.

Both the model types (weak or strong) and the fault types (intermittent or non-intermittent) change the way we approach the MO-MBD problem. An algorithm that assumes that a fault consistently appears does not apply to a system with intermittent faults, and therefore we need to develop different solutions based on the conditions of the problem. **The first contribution** of this paper is by exploring the four possible combinations: intermittent+SFM, non-intermittent+SFM, intermittent+WFM, and non-intermittent+WFM.

Several approaches were proposed for model-based diagnosis with multiple observations. Some proposed a conflict-directed approach [6,7,8], generating conflicts for each observation and merging them together. Others proposed a hierarchical approach-based structural abstraction and compilation to d-DNNF [9]. Lastly, a SAT-based approach was implemented [10]. Encouraged by recent success of model-based diagnosis algorithms for classical diagnosis that are based on compilation to Boolean satisfiability (SAT) [11,12], we investigate in this work a SAT-based approach for MO-MBD that considers both the intermittent non-intermittent axis as well as the SFM WFM axis.

**A second contribution** of the paper is by presenting two SAT-based algorithms for solving the MO-MBD problem in the intermittent+WFM configuration: (1) one-SAT: solving all the observations at once, or (2) divide-and-join: solving each observation separately and combing the diagnoses in a way that is consistent with all of them. The first way merges all the observations into one, without losing any information, and compiles the problem into a single formula. The second, compiles each observation to its own SAT formula and then joins the resulting diagnoses. In this paper we present these two methods in detail for the intermittent+WFM configuration, but they could be adapted to each one of the other configurations.

Experimental evaluation of the intermittent+WFM configuration on known benchmarks demonstrates pros and cons of each algorithms. divide-and-join is faster for cases with fewer abnormal behaviors, while one-SAT is more appropriate for cases in which there are many abnormal behaving components.

The paper is structured as follows. First, background and formal definition of MBD and MO-MBD is given, as well as related work. Then, we characterize different assumptions about the behavior of components in a MO-MBD problem. Following, we introduce the two SAT-based algorithms we propose and evaluate them empirically. Lastly, we conclude and discuss future work.

## 2. Background and Related Work

In this section, we present the fundamentals of model-based diagnosis (MBD) and related works.

### 2.1. Model-Based Diagnosis

An MBD problem is defined by a tuple 〈SD,COMPS,OBS〉, where SD is a model of the diagnosed system, COMPS is the set of system components, and OBS is the observed behavior of the system (e.g., the observed inputs and outputs of the system). The system model SD specifies for every component a set of possible assumptions about its behavior. These assumptions are called *behavior modes* and SD contains at least one behavior mode per component that specifies its normal behavior. We say that a component *c* has a strong-fault model (SFM) if SD contains some information about how *c* behaves when it is faulty, i.e., when its behavior mode is not the normal one. Otherwise, we say that *c* has a weak-fault-model (WFM). We denote the normal behavior mode and the set of all other behavior modes of *c* by ok and $F_{c}$, respectively. The behavior modes in $F_{c}$ are also referred to as *fault modes*. Please note that if *c* has a WFM then $F_{c}$ contains a single-fault mode—unknown.

A solution to an MBD problem is a *diagnosis*. A diagnosis is a set of assumptions about the behavior of the system components that form a plausible explanation of the observed behavior. To define a diagnosis formally, we associated with every component *c* a variable $h_{c}$, referred to as the *health variable*, whose value is the behavior mode of *c* when the system was observed. A *health assignment* is an assignment of behavior modes to all the health variables. In logical terms, a health assignment is a conjunction of propositional literals of the form $h_{c}=m$ where $m\in F_{c}\cup \left\{ok\right\}$. If the health assignment in which all components are assigned their normal behavior mode is inconsistent with SD and OBS then at least one component must be faulty, i.e., there exists at least one component *c* such that $h_{c}\in F_{c}$.

**Definition** **1** (Diagnosis). *A health assignment ω is called a diagnosis if SD∧OBS∧ω is satisfiable.*

The number of diagnoses can be very large. Therefore, it is common in the MBD literature to focus on finding only the *subset-minimal* (SM) diagnoses or only the *minimal-cardinality* (MC) diagnoses. Both notions—SM diagnoses and MC diagnoses—are explained next. For a diagnosis ω we denote by $\omega^{-}$ the components in ω assigned to a faulty behavior mode. The size of $\omega^{-}$ is referred to as the *cardinality* of ω, denoted by |ω|. A diagnosis is called an SM diagnosis if there is no other diagnosis $\omega^{\prime }$ such that $\omega^{\prime -}\subset \omega^{-}$. A diagnosis is called an MC diagnosis if there is no other $\omega^{\prime }$ such that $|\omega^{\prime }\left|<|\omega \right|$. Many methods to find diagnoses have been proposed, including conflict-directed [1,13,14], compilation-based [15,16], SAT-based [11], and distributed approaches [17,18]. The General Diagnosis Engine (GDE) [19] is a notable example of a conflict-directed MBD algorithm that has been applied in numerous domains. GDE, and other conflict-directed MBD algorithms, work by identifying conflicts, which are partial health assignments that are inconsistent with SD and OBS, and then finding diagnoses by considering hitting sets of the identified conflicts. Finding conflicts is in itself a difficult problem, and GDE solves it by using an Assumption-based Truth Maintenance System (ATMS) [19,20]. SATbD [11] is an example of a SAT-based MBD algorithm. SAT-based MBD algorithm work by reducing an MBD problem to one or more Boolean satisfiability (SAT) problems, which are then solved with an off-the-shelf SAT solver. GDE, SATbD, and the other methods mentioned above assume that OBS consists of a single observation. Next, we present a literature review about papers that consider multiple observations.

### 2.2. Related Work

De Kleer proposed two extensions to GDE to support MBD with multiple observations, one for intermittent faults [7] and one for non-intermittent faults [6]. In the GDE extension for intermittent faults, De Kleer defined a function g(c) to represent the conditional probability that the component *c* is behaving correctly although it is faulty [7]. Essentially, if g(c)=1 then *c* is persistently faulty, and if g(c)=0 then *c* does not exhibits faulty behavior. A value between 0 and 1, indicates an intermittent fault. Applying this GDE extension is a challenge since usually g(c) is not known a priori. However, methods have been proposed to estimate g(c) using a maximum likelihood estimation approach [21,22,23,24].

In the GDE extension for non-intermittent faults [6], clauses are added to the ATMS to represent the different times of observations. This enables the components to be represented not only by their input and output, but also by the combination of their inputs and outputs and time. Additional clauses were added to specify that (1) Time cannot be the same for two observations, (2) Time cannot exist on its own, and (3) the same component must act the same across all observations. These clauses ensure non-intermittency.

Ignatiev et al. [25] proposed a MaxSat approach to compute MS diagnoses in a sequential diagnosis setting, where multiple observations are given as input. They do not, however, propose a method for returning MC diagnoses, and do not explicitly address the distinctions between intermittent and non-intermittent and between SFM and WFM. As we show in this work, these distinctions are important. Others proposed a conflict-directed approach for sequential diagnosis [6,7,8], where conflicts are generated for each observation independently, and then merged. Others proposed a hierarchical approach based on structural abstraction and a compilation to d-DNNF [9]. None of these prior works focus on returning MC diagnoses or adopt a SAT-based approach.

Cai et al. [26] proposed to use a Bayesian network to handle diagnosis of intermittent faults and to return the most probable diagnosis. Boaziat et al. [27] proposed an MBD approach to solve MBD with intermittent faults in discrete event systems. Recently, Zhou et al. [28] reviewed different model-based and data-driven methods to find the most probable diagnosis in dynamic systems with intermittent faults. Gómez et al. [29] proposed an approach for diagnosis in the presence of intermittent faults that applies Bayesian reasoning to distinguish between different types of faults [29]. During the diagnosis process, faults are not only found but also classified as either intermittent or transient faults. The diagnosis process combines then two methods: window-based diagnosis and Bayesian reasoning.

In general, most previous work focused on intermittent faults. The contributions of this paper include formally defining intermittent and non-intermittent faults for weak and strong-fault models. Also, we propose diagnosis algorithms to solve the configuration of intermittent fault in weak-fault model, focusing on how computing MC diagnoses using variations of SAT-based MBD approach, which has been proven as efficient for diagnosis problems [11].

Our work is related to *sequential diagnosis*, which is a diagnosis problem in which observations are generated and given the diagnostic system sequentially. Most previous work on sequential diagnosis focused on which observation to generate to reduce the number of diagnoses [9,30,31,32,33], or to learn more about the probabilities of components to be faulty [34]. Our work can be used by sequential diagnosis algorithm to allow them to process faster the sequence of observations they receive.

Our work is also related to prior work on modeling intermittent and non-intermittent faults. Breuer is one of the first to model intermittent faults, proposing a Markov model for this purpose [35]. Based on Breuer’s model, Koren suggested a method to converting single-fault intermittent diagnosis of combinational logic to dynamic programming, by building a nearly minimal sequential decision tree that minimizes the average number of distinguishing tests required to locate a fault [36].

## 3. MBD with Multiple Observations

In this work we focus on an MBD problem with multiple observations, taken in different time steps. Let *T* = $\left\{t_{1},\ldots ,t_{n}\right\}$ be the set of time points in which the system was observed, and ${\mathrm{OBS}}_{t_{i}}$ is the observation at time point $t_{i}$. The *multiple observation MBD* (MO-MBD) problem is defined by a tuple $\langle \mathrm{SD},\mathrm{COMPS},T,{\left\{{\mathrm{OBS}}_{t}\right\}}_{t\in T}\rangle$, where SD and COMPS are the system description and set of components as above. Following Raiman et al. [6], every time step t∈T is called an *observation time*.

A solution to a MO-MBD is also a diagnosis, but a diagnosis in the context of MO-MBD is an assumption about the behavior of the system components that is a plausible explanation for *all* the observations. Formally, we associate with every pair of component *c* and observation time *t* a *timed health variable* $h_{c,t}$. The domain of $h_{c,t}$ is the behavior modes of component *c* and its value represents the behavior mode of *c* at observation time *t*. A *timed health assignment* is an assignment of behavior modes to all the timed health variables.

**Definition** **2** (Diagnosis for MO-MBD). *A timed health assignment ω is a diagnosis of a MO-MBD problem iff $\mathrm{SD}\wedge {⋀}_{t\in T}{\mathrm{OBS}}_{t}\wedge \omega$ is satisfiable.*

As was the case in the classical MBD problem, the number of diagnoses for a MO-MBD problem can be very large. Therefore, a form of minimality criteria over MO-MBD diagnoses is needed to focus the problem solver. To this end, we define the cardinality of a MO-MBD diagnosis as the number of components that are assumed faulty in at least one observation time. Formally, the cardinality of a diagnosis ω, denoted by

|ω|=|{c|c∈COMPSand∃t∈Th(c,t)≠ok}|

Our goal in this work is to find MC diagnoses for the MO-MBD problem.

## 4. Intermittent and Non-Intermittent Faults

Definition 2 embodies a fundamental difference between MBD and MO-MBD: in MO-MBD a component may, in general, output different values for the same input values in different observation times. This can be due to changes in its state between observation times, and due to some un-modeled aspect of the environment. This is referred to as an *intermittent* behavior mode. In some cases, however, it is reasonable to assume that the component’s behavior is consistent over time, i.e., that for the same input the component will generate the same output. This is referred to as a *non-intermittent* behavior mode.

Whether faults are intermittent or not is domain-dependent. As an example, software components are notorious for their intermittent behavior, due to the computer’s multi-tasking nature and dependence on many external aspects (e.g., network speed). By contrast, it is reasonable to assume a non-intermittent behavior from a leaking valve in a hydraulic system, since it is expected to always leak when flooded with fluid.

We follow Raiman et al. [6] and formally define a component with a non-intermittent behavior as follows. Let in(c,t) and out(c,t) be the values inputted to and outputted by a component *c* at observation time *t*.

**Definition** **3** (Non-Intermittent Behavior). *A component c is said to have a non-intermittent behavior mode iff there exists a function F such that*

∀t∈T:F(in(c,t))=out(c,t)

A component with an intermittent behavior mode is a component for which such a function *F* may not exist.

### 4.1. Between Fault Modes and Intermittency

A component may have an intermittent (Int) or a non-intermittent (NotInt) behavior mode, and also it may have a strong-fault model (SFM) or a weak-fault model (WFM). The first observation of this work is that each of the resulting four combinations (Int+SFM, NotInt+SFM, Int+WFM, and NotInt+WFM) is possible. Next, we formally define each of these combinations.

#### 4.1.1. Int+WFM

This is the least constrained assumption one can have on a component. There is no constraint on the abnormal behavior of the component and the component may behave differently in different observation times. Let $\phi_{c}$ be a function describing the healthy behavior of component *c*, and let h(c,t) be a predicate that is true iff component *c* follows its healthy behavior at observation time *t*, i.e., $h\left(c,t\right)\equiv \left(h_{c,t}=ok\right)$. A component that is Int+WFM is defined as follows:

(1) $$\forall t\in T:h\left(c,t\right)\to (out\left(c,t\right)=\phi_{c}\left(in\left(c,t\right)\right))$$

That is, we can expect the component to follow its intended behavior if it is healthy, but if it is not healthy then we have no knowledge about how it will be behave.

#### 4.1.2. NotInt+WFM

Although the faulty component’s behavior is not specified (Equation (1)), a NonInt+WFM component’s faulty behavior is constrained to be consistent along observations (Definition 3). Thus, the formal Definition of a NonInt+WFM component is a mixture of Definition 3 and Equation (1).

(2) $$\begin{array}{c} \exists \phi_{c}^{F}\;s.t.\;\forall t\in T:\;\left(h\left(c,t\right)\to (out\left(c,t\right)=\phi_{c}\left(in\left(c,t\right)\right))\right)\wedge \left(\neg h\left(c,t\right)\to (out\left(c,t\right)=\phi_{c}^{F}\left(in\left(c,t\right)\right))\right) \end{array}$$

In words, if *c* is a NonInt+WFM component, then in addition to its behavior when it is healthy, we also know that there if it faulty then there exists a fault model $\phi_{c}^{F}$ that defines its behavior in all observations. Please note that while we do not know this fault model ($\phi_{c}^{F}$), we can expect it to be consistent across observations.

#### 4.1.3. Int+SFM

Here, we know the possible way in which the component may behave when faulty (its fault modes). However, the component may act differently in different observation times, i.e., switch between a healthy behavior and a faulty behavior (Here we describe a strong form of intermittency, where a component may have different fault modes in different observation times. One can also envision a weaker form of intermittency, in which a faulty component is associated with a single-fault behavior mode, but can act normally in some observations. Addressing this is variant is left for future work). Let $\phi_{c}^{m}$ denote the function describing the behavior of component *c* when in mode *m*. f(c,t,m) is a predicate that is true iff component *c* followed the behavior of mode *m* at observation time *t*. An Int+SFM component is defined as follows:

(3) $$\begin{array}{c} \forall t\in T:\;\left(h\left(c,t\right)\to (out\left(c,t\right)=\phi_{c}\left(in\left(c,t\right)\right))\right)\wedge \underset{m\in \left\{ok\right\}\cup F_{c}}{⋀}\left(f\left(c,t,m\right)\to (out\left(c,t\right)=\phi_{c}^{m}\left(in\left(c,t\right)\right))\right) \end{array}$$

In addition, we must define that a component *c* at time *t* has exactly one behavior mode $m\in \left\{ok\right\}\cup F_{c}$.

The above formal definition means that if *c* is a Int+SFM component, then we know that it behaves according to one of its behavior modes—healthy of faulty. It may behave according to one fault mode in one observation and according to a different fault mode in another.

#### 4.1.4. NotInt+SFM

This is the most constrained behavior mode. A NonInt+SFM component must behave consistently throughout the observations, and its faulty behavior is specified as one of the fault modes in *M*: The formal definition is the same as in the Int+SFM case (Equation (3)), but with an additional constraint to represent the non-intermittent behavior:

(4) $$\begin{array}{c} \forall t,t^{\prime }\in T:\left(h\left(c,t\right)↔h\left(c,t^{\prime }\right)\right)\wedge \underset{m\in \left\{ok\right\}\cup F_{c}}{⋀}\left(f\left(c,t,m\right)↔f\left(c,t^{\prime },m\right)\right) \end{array}$$

In words, if *c* is a NonInt+SFM component, then it follows exactly one behavior mode in all observations.

Table 1 summarizes the different configurations.

## 5. Finding Diagnoses

Next, we propose algorithms for solving a MO-MBD problem, focusing on the Int+WFM configuration. As mentioned in the introduction the approaches can be extended to the other configurations. Raiman et al. [6] proposed a conflict-directed approach for solving an MBD problem with multiple observations. They extracted *conflicts* from each observation, and every minimal hitting set of all the conflicts is considered to be a diagnosis.

Following the recent success of SAT-based solvers for the classical, single-observation, MBD problem [11], we explore a different approach that is also based on a SAT compilation. We first briefly describe the SAT-based approach for solving the classical MBD problem, and then explain two ways to extend it for MO-MBD problem.

### 5.1. SAT-Based MBD Algorithm

A SAT solver is an algorithm that accepts as input a Boolean formula and outputs a satisfying assignment of that formula, if such exists, or false otherwise. Compile an MBD problem to a SAT problem, i.e., to a Boolean formula, has been done in prior work [11,37]. Briefly, clauses are defined for every component specifying that a component has exactly one behavior mode. For every component *c* and behavior mode *m* we add clauses to specify its behavior ($\phi_{c}^{m}$). In a WFM, we can define a single clause for a component, specifying that if it is healthy then it will act normally. In addition, there is a clause for every observation, specifying the values that were observed. The variables of this Boolean formula include the health variables $h_{c}$, and the values of these variables in a satisfying assignment are exactly a diagnosis.

The process of finding MC diagnoses starts with finding the cardinality of the MC diagnoses. This is done by adding clauses that enforce a cardinality constraint [38,39] over the number of components assigned a faulty mode. This cardinality bound is initially set to some upper bound of the MC and it is then iteratively decreased if the resulting formula is satisfiable. When the formula is not satisfiable, it indicates that the previous cardinality bound is the MC. Then, the cardinality bound is set to the found MC, and a SAT solver is used to return all satisfying assignments, which are exactly all the MC diagnoses. For more details on this process see Metodi et al. [11].

**Figure 1:**
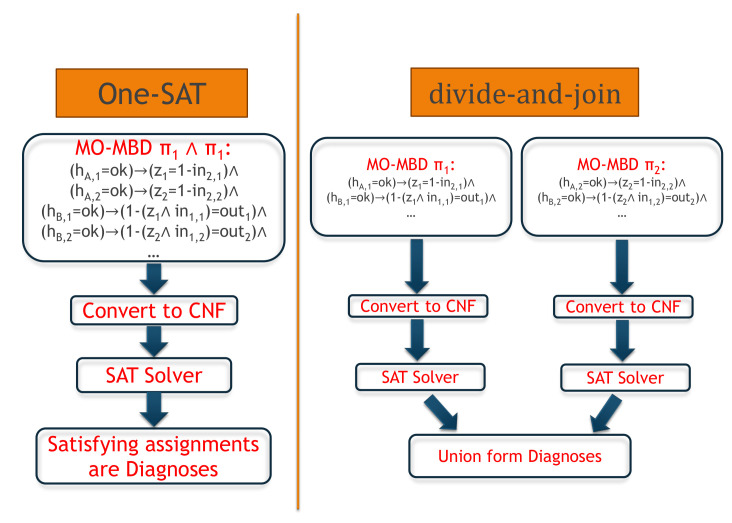
A diagram of the proposed two approaches.

In this work we propose two approaches. In the first approach, One-SAT, we represent the constraints of all the MO-MBD problems together and convert these constraints to CNF, and then find the diagnoses by a SAT solver. In the second approach, divide-and-join, we represent the constraint of each MO-MBD problem separately, convert these constraints to CNF, and find the diagnoses by a SAT solver. Then we combine the solutions of the MO-MBD problems. For the sake of clarity, the flow of each approach is depicted in Figure 1 draws the flow of each approach. The first approach is presented in Section 5.2, and the second approach is presented in Section 5.3.

### 5.2. One Formula for Multiple Observations

The first way we propose to encode the MO-MBD problem is to construct a single Boolean formula that encodes the knowledge from all the observations. Doing this is under the assumption that components fail intermittently (recall that we focus on the Int+WFM) is simple, since the observations can be encoded independently to a Boolean formula and we can just merge them together. Please note that all the variables that represent the internal state of the system must be duplicated, to allow them to receive different values for different observations. A similar approach was proposed in other contexts, such as diagnosing Linear Temporal Logic [40] and verifing diagnosability [41].

**Figure 2:**
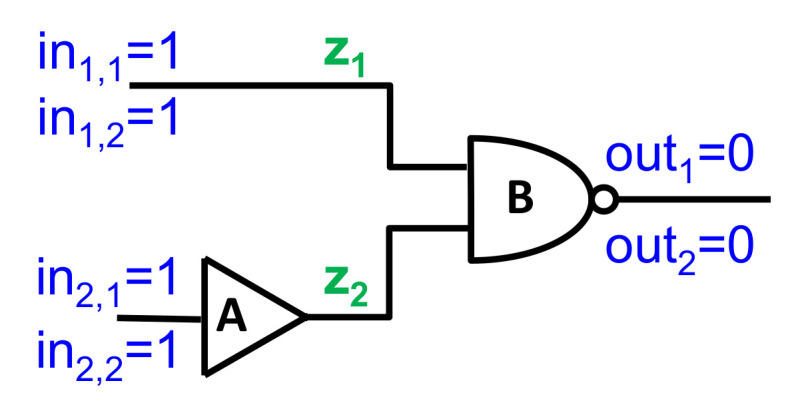
An example of a system with two observations.

To illustrate this approach, consider the simple example depicted in Figure 2. For i=1,2 and j=1,2, we denote by $out_{j}$ and $in_{i,j}$ the output and the $i^{th}$ input, respectively, of the $j^{th}$ observation. Similarly, the variable *z* represents the output of component *A*, where $z_{1}$ and $z_{2}$ are the values at observation times 1 and 2, respectively. The resulting Boolean formula for Figure 2 is given in Equations (5)–(10). Equations (5)–(8) describe the normal behavior of components *A* and *B*. Please note that since we focus on WFM, we describe only the normal behavior of these gates. We do not describe the faulty behavior, which could provide any output. Equation (9) describes the observations’ inputs, and Equation (10) describes the observations’ outputs.

(5) $$\begin{array}{cc} (h_{A,1}=ok)\to & \left(z_{1}=1-in_{2,1}\right) \end{array}$$

(6) $$\begin{array}{cc} \wedge (h_{B,1}=ok)\to & \left(1-\left(z_{1}\wedge in_{1,1}\right)=out_{1}\right) \end{array}$$

(7) $$\begin{array}{cc} \wedge (h_{A,2}=ok)\to & \left(z_{2}=1-in_{2,2}\right) \end{array}$$

(8) $$\begin{array}{cc} \wedge (h_{B,2}=ok)\to & \left(1-\left(z_{2}\wedge in_{1,2}\right)=out_{2}\right) \end{array}$$

(9) $$\begin{array}{cc} \wedge in_{1,1}=1\wedge in_{1,2}=1 & \wedge in_{2,1}=1\wedge in_{2,2}=1 \end{array}$$

(10) $$\begin{array}{cc} \wedge out_{1}=0 & \wedge out_{2}=0 \end{array}$$

To find MC diagnoses, we also add a set of clauses to constrain the cardinality of the returned diagnoses. This is done by defining a health predicate $H_{c}$ that is not timed (in contrast to the time-health variable $h_{c,t}$) for every component *c*, such that $H_{c}$ is true iff *c* is assumed to behave normally in all observations. Thus, each such health predicate is associated with the following clause: $H_{c}↔{⋀}_{t\in T}\left(h_{c,t}=ok\right)$. For the example in Figure 2 we add the following clauses for $H_{A}$ and $H_{B}$.

(11) $$\begin{array}{cc} H_{A}↔ & \left(\left(h_{A,1}=ok\right)\wedge \left(h_{A,2}=ok\right)\right) \end{array}$$

(12) $$\begin{array}{cc} H_{B}↔ & \left(\left(h_{B,1}=ok\right)\wedge \left(h_{B,2}=ok\right)\right) \end{array}$$

The process of finding all MC diagnoses is similar to the process described earlier for the classical MBD problem. A cardinality constraint is added to the Boolean formula that constrains these number of health predicates that are set to false. The constraint starts with some upper bound UB on the cardinality of the MC diagnoses, and we decrease UB iteratively (one by one) until the resulting Boolean formula is not satisfiable. This indicates that the previous value of UB is the minimal cardinality, and we set the cardinality constraint to this value to find all MC diagnoses. We consider true (or ok) assignment as 1 and false as 0, and thus, the cardinality constrain will be:

(13) $$\begin{array}{c} \sum_{i=A}^{\left|COMPS\right|}\neg H_{i}=UB \end{array}$$

A diagnosis includes the components have the health value $\neg H_{i}$. We then repetitively ask the SAT solver to return a different assignment in size UB, until there are no more possible assignments. We call the above algorithm, which compiles the MO-MBD problem into a single Boolean formula, the one-SAT algorithm.

### 5.3. Joining Diagnoses of Multiple Observations

Encoding the knowledge about all observations into a single Boolean formula allows using the full power of modern SAT solvers. However, the size of the resulting encoding grows linearly with the number of observations. This can become a big computational problem since the worst-case runtime complexity of SAT solvers is exponential in the size of the encoding. Next, we propose an approach that solves each observation independently, and then joins the resulting set of diagnoses into a single diagnosis for the given MO-MBD problem.

Let $\Pi =\langle \mathrm{SD},\mathrm{COMPS},T,{\left\{{\mathrm{OBS}}_{t}\right\}}_{t\in T}\rangle$ be a MO-MBD problem. We define $\Pi_{i}=\langle \mathrm{SD},\mathrm{COMPS},{\mathrm{OBS}}_{i}\rangle$ as the (single-observation) MBD problem that uses the same system model and components as Π but considers only observation *i*. Let Ω(Π) and $\Omega (\Pi_{i})$ denote the set of all diagnoses for the MO-MBD and MBD problems Π and $\Pi_{i}$, respectively. Since we focus on WFM a component in a diagnosis is either assumed to be healthy (ok) or not. Thus, we can represent a diagnosis as the set of components that are assumed to be faulty instead of a health assignment (which maps every component—healthy and faulty—to its behavior mode). For convenience of notation, we do so hereinafter, and thus every element in Ω(Π) and $\Omega (\Pi_{i})$ is simply a set of components. Slightly abusing standard relational algebra terminology, we define the *join* operation between two sets of diagnoses $\Omega_{i}$ and $\Omega_{j}$, denoted $\Omega_{i}⋈\Omega_{j}$, as follows:

$$\Omega_{i}⋈\Omega_{j}=\left\{\omega_{i}\cup \omega_{j}|\omega_{i}\in \Omega_{i},\omega_{j}\in \Omega_{j}\right\}$$

Since we assume that faults are intermittent, then

(14) $$\Omega \left(\Pi \right)=\Omega \left(\Pi_{1}\right)⋈\Omega \left(\Pi_{2}\right)⋈\ldots ⋈\Omega \left(\Pi_{n}\right)$$

Therefore, we can find all diagnoses for the MO-MBD problem Π by solving the set of MBD problems $\Pi_{1},\ldots \Pi_{n}$ individually, and joining the results. We call this algorithm divide-and-join.

The worst-case runtime of current complete SAT solvers is exponential in the size of the Boolean formula they are given. Thus, solving the *n* Boolean formulae that represent the MBD problems $\Pi_{1},\ldots ,\Pi_{n}$ has a worst-case runtime that is exponentially smaller than solving the single Boolean formula for Π, as the encoding for Π is *n* times larger than that of $\Pi_{i}$.

However, and even worse than the runtime of the one-SAT algorithm due to the runtime of the join operation. Under a naive implementation, the join operation requires running over the cross product of all the diagnoses sets $\Omega \left(\Pi_{1}\right),\ldots ,\Omega \left(\Pi_{n}\right)$, thus, requiring runtime that is exponential in the number of observations. It is not obvious, at least to the authors, if there is a more efficient way to compute this join (notice its difference from standard relational algebra join, which can be implemented more efficiently). Moreover, the number of diagnoses returned by each MBD problem $\Pi_{i}$ can be exponential in the number of components. Lastly, each activation of the SAT solver incurs some overhead, which is incurred *n* times for divide-and-join while this overhead is only incurred once for one-SAT. Indeed, as we show in the experimental results, there is no universal winner and which algorithm is more efficient depends on various domain properties.

#### 5.3.1. Finding Minimal Diagnoses

The divide-and-join algorithm was described above for finding all diagnoses, which, as discussed earlier, can be prohibitively large. It turns out that using divide-and-join for finding only SM diagnoses is straightforward: simply find all SM diagnoses for $\Pi_{1},\ldots ,\Pi_{n}$ and join the results. The correctness of this approach is a direct result of the following Lemma (proof is omitted due to space constraints).

**Lemma** **1.** *If $\Omega^{SM}\left(\Pi \right)$ and $\Omega^{SM}\left(\Pi_{i}\right)$ are the set of SM diagnoses for* Π *and $\Pi_{i}$, respectively, then* $\Omega^{SM}\left(\Pi \right)=\Omega {\left(\Pi_{1}\right)}^{SM}⋈\Omega {\left(\Pi_{2}\right)}^{SM}⋈\ldots ⋈\Omega {\left(\Pi_{n}\right)}^{SM}$

Lemma 1 does not carry over to MC diagnoses, i.e., the join of all MC diagnoses for $\Pi_{1},\ldots ,\Pi_{n}$ may not contain all MC diagnoses for Π and may contain diagnoses that are not MC diagnoses of Π. Formally, if $\Omega^{MC}\left(\Pi \right)$ and $\Omega^{MC}\left(\Pi_{i}\right)$ are the set of MC diagnoses for Π and $\Pi_{i}$, respectively, it may happen that:

$$\Omega^{MC}\left(\Pi \right)\neq \Omega {\left(\Pi_{1}\right)}^{MC}⋈\Omega {\left(\Pi_{2}\right)}^{MC}⋈\ldots ⋈\Omega {\left(\Pi_{n}\right)}^{MC}$$

As an example, consider a MO-MBD problem with two observations, such that:

$$\begin{array}{cc} \Omega^{SM}\left(\Pi_{1}\right)= & \left\{\{A,B\}\right\}\\ \Omega^{SM}\left(\Pi_{2}\right)= & \left\{\{E,F\},\{A,B,C\}\right\} \end{array}$$

Then $\Omega^{MC}\left(\Pi_{1}\right)=\left\{\left\{A,B\right\}\right\}\;\mathrm{and}\;\Omega^{MC}\left(\Pi_{2}\right)=\left\{\left\{E,F\right\}\right\}$ The join $\Omega^{MC}\left(\Pi_{1}\right)⋈\Omega^{MC}\left(\Pi_{2}\right)$ is the diagnosis ω={A,B,E,F} with cardinality 4. However, the MC diagnosis for Π is actually $\omega^{\prime }=\left\{A,B,C\right\}$ with cardinality 3. Therefore, divide-and-join cannot find MC diagnoses without further adaptations. To this end, we introduce divide-and-join-MC, a modified version of divide-and-join able to find all MC diagnoses.

#### 5.3.2. Finding MC Diagnoses with Divide-and-Join

Next, we describe how divide-and-join can be modified to find MC diagnoses. We call this algorithm Divide-and-Join-MC (described in Algorithm 1). To properly explain divide-and-join-MC, we require the following terminology. Let mc and $mc_{i}$ be the cardinality of the MC diagnoses for the MBD problems Π and $\Pi_{i}$, respectively. Also, let $\Omega^{n}\left(\Pi \right)$ and $\Omega^{n}\left(\Pi_{i}\right)$ be the set of all SM diagnoses of cardinality *n* or less for Π and $\Pi_{i}$, respectively. A *cardinality bounds vector* is an *n*-ary vector $b=\langle b_{1},\ldots ,b_{n}\rangle$ such that for every *i* in the range [1,n] it holds that $b_{i}\geq mc_{i}$.

We say that a cardinality bounds vector b is *MC-sufficient* iff

$$\Omega^{MC}\left(\Pi \right)\subseteq \Omega^{b_{1}}\left(\Pi_{1}\right)⋈,\ldots ,⋈\Omega^{b_{n}}\left(\Pi_{n}\right)$$

Finding an MC-sufficient cardinality bounds vector is important since it proving that all MC diagnoses of Π have been found. Finally, for a set of diagnoses *X*, we define MC(X) as the cardinality of the diagnoses that has the smallest cardinality in *X*, i.e., $MC\left(X\right)=\min_{\omega \in X}\left|\omega \right|$.
**Algorithm 1:**Divide-and-Join-MC
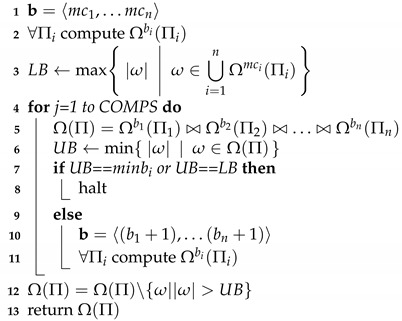


Divide-and-Join-MC starts by initializing a cardinality bounds vector b by the cardinality of the MC diagnoses of the individual MBD problems, i.e., initially $b=\langle mc_{1},\ldots ,mc_{n}\rangle$ (line 1). In every iteration, the algorithm computes for every problem $\Pi_{i}$ all the SM diagnoses of cardinality equal to or lower than $b_{i}$, this initially done in line 2 and then again in line 11. Then, in line 7, the algorithm attempts to prove that b is an MC-sufficient cardinality bounds vector, by inspecting whether the join of these sets of diagnoses is the set of all MC diagnoses of Π. If the algorithm can prove that b is an MC-sufficient cardinality bounds vector - it terminates, returning all diagnoses in the join that have the smallest cardinality. Otherwise, the cardinality bounds vector is incremented (adding one to all its elements), and the process continues (lines 9–10).

The key question is how to prove that a cardinality bounds vector is MC-sufficient. For this, we provide the following simple rule: if UB is an upper bound on the MC of Π then b=〈UB,…,UB〉 is MC-sufficient. The challenge is how to find a low enough UB. For every cardinality bounds vector b, it holds that every diagnosis ω in $\Omega^{b_{1}}\left(\Pi_{1}\right)⋈,\ldots ,⋈\Omega^{b_{n}}\left(\Pi_{n}\right)$ is a diagnosis of Π and thus |ω| is an upper bound on mc. Thus, every iteration of the algorithm can provide a better UB. Concretely, the divide-and-join algorithm starts with a cardinality bounds vector $b=\langle mc_{1},\ldots ,mc_{n}\rangle$ and in every iteration obtains the current UB (by computing all diagnoses for b and joining them). If $UB=\min_{i}b_{i}$ all MC diagnoses have been found. Otherwise, a new iteration starts with every element in b incremented by one.

To demonstrate the algorithm, assume a MO-MBD problem with two observations. The MC diagnoses are:

$$\begin{array}{cc} \Omega^{MC}\left(\Pi_{1}\right)= & \left\{\{A\},\{E\}\right\}\\ \Omega^{MC}\left(\Pi_{2}\right)= & \left\{\{B,C\},\{C,D\}\right\} \end{array}$$

and b=〈1,2〉 (1 is the minimal cardinality of $\Omega^{MC}\left(\Pi_{1}\right)$ and 2 is the minimal cardinality of $\Omega^{MC}\left(\Pi_{2}\right)$). LB will be equal to 2 since it is the max cardinality in b. We then compute Ω(Π) and UB which is the minimal cardinality over the diagnoses in Ω(Π).

Ω(Π)={{A,B,C},{E,B,C},{A,C,D},{E,C,D}}

*UB* = 3

Now, since UB≠1 (which is the minimal cardinality in **b**) and UB≠2 (which is LB) we continue and add 1 to every cardinality in **b**, i.e., b=〈2,3〉. We compute the diagnoses of each observation with this cardinality and get:

$$\begin{array}{cc} \Omega^{2}\left(\Pi_{1}\right)= & \left\{\{A,B\},\{E,C\}\right\}\\ \Omega^{3}\left(\Pi_{2}\right)= & \left\{\{A,B,C\},\{A,F,B\}\right\} \end{array}$$

These diagnoses are added to the diagnoses of each observation that were computed in all previous steps. That means that when we do the join process, we join the previous diagnoses with the new.

Ω(Π)={{A,B,C},{E,B,C},{A,C,D},{E,C,D},{A,F,B},{A,B,C,E},{A,F,B,E}, {A,F,B,E,C}}

*UB* = 3

At this point we will still not reach the condition for halting but after increasing all the cardinalities in **b** and computing the new diagnoses for each observation There are still no match so we increase the cardinality of both diagnoses again. This will result in $UB=minb_{i}$ since UB never grows but $minb_{i}$ will be now equals to 3. Then we will compute again all the join result and return the ones with the cardinality of 3.

For a cardinality bounds vector b, let $b_{min}=\min_{i}b_{i}$, ${⋈}_{b}\left(\Pi \right)=\Omega^{b_{1}}\left(\Pi_{1}\right)⋈\ldots ⋈\Omega^{b_{n}}\left(\Pi_{n}\right)$, and let $UB_{b}=\min\{|\omega ||\omega \in {⋈}_{b}\left(\Pi \right)\}$.

**Theorem** **1.** *For every cardinality bounds vector b if $b_{min}\leq UB_{b}$ then $b_{min}$ is a lower bound on mc.*

**Proof.**  Assume by negation that $b_{min}>mc$, so there exists a diagnosis ω whose cardinality is lower than $b_{min}$. Please note that $\omega \in {⋈}_{i=1}^{n}$ due to Equation (14), and therefore, there exist $\omega_{1},\ldots ,\omega_{n}$, where $\omega_{i}\in \Omega \left(\Pi_{i}\right)$ for every *i*, and $\omega ={⋃}_{i=1}^{n}\omega_{i}$. Thus, the cardinality of ω is not smaller than the cardinality of each of the constituents $\omega_{i}$. Therefore, for every *i* it holds that $\omega_{i}\in \Omega^{b_{i}}\left(\Pi_{i}\right)$, since $b_{i}\geq b_{min}>|\omega |\leq |\omega_{i}|$, and consequently $\omega \in {⋈}_{b}\left(\Pi \right)$, reaching the desired contradiction. □

## 6. Empirical Evaluation

We evaluated the one-SAT and divide-and-join-MC algorithms on Boolean circuit systems. Specifically, we experimented on the 74181 (with 65 components) and 74283 (36 components) systems from the 74XXX [42] benchmark suite, and the c432 (160 components) and c880 (383 components) systems from the ISCAS-85 [43] benchmarks suite.

The details (number of components and number of inputs and outputs) of the chosen systems are given in Table 2. The systems 74XXX [42] are described in the first two rows, and additional two systems of ISCAS-85 [43] are described in the following two rows. Although these Boolean circuits are lacking some aspects of real-world challenges, they have been used for experiments in many previous MBD papers [11,14,44,45,46,47]. However, our approach can be applied directly in any setting where the components are described by propositional formulae. Boolean circuits are just one straightforward example where this is obvious and where the community has focused attention. Other examples are: (1) formulating software components as propositional formulae and apply an MBD algorithm to find bugs [24,46,47,48]; (2) modeling robots in a multi-robot system and diagnose the violation of coordination constraints among robots [49,50]; (3) modeling finite domain constraints and diagnose inconsistent constraint sets [51,52].

To the best of our knowledge, there is no standard set of observations for MO-MBD or sequential MBD problems. Thus, we generated for each of these systems random MO-MBD problems with 2, 4, 6, and 10 observations, as follows. First, faults are injected to 2–4 randomly selected components. Fault injection to gates in such systems is standard in the literature [9,44]. Then, for every observation we generate random input values and propagate them in the system. The faulty components behave abnormally—i.e., negate the normal output—with probability $p_{int}$, where $p_{int}$ is a parameter. $p_{int}$ controls the “intermittency” of the components, where $p_{int}=0$ means faulty component always behave normally, and $p_{int}=1$ means that the faulty component will always behave abnormally. We experimented with a $p_{int}$ value of 0.3,0.5,0.7,0.85, and 1. For each configuration of system, number of faults, number of observations, and $p_{int}$, we generated 15 different MO-MBD problems.

Each problem was solved with one-SAT and with divide-and-join, and the runtime required to find the First MC diagnosis and the runtime required to find All MC diagnoses were both recorded. Please note that while parts of the divide-and-join algorithm could be parallelized, we used in all our experiments a single core to allow a fairer comparison. Both algorithms were run on a single core and we measured the runtime of finding the first MC diagnosis and all MC diagnoses. A timeout of 15 min was imposed. Most problem instances were solved before reaching this timeout. Specifically, one-SAT solved 75%, 78%, 64%, and 68% of the instances of systems 74182, 74181, c432, and c880, respectively, and divide-and-join-MC solved 75%, 78%, 68%, and 61% of the instances, for the same set of systems. The runtime results presented below are averages over instances that were solved by both algorithms.

**Figure 3:**
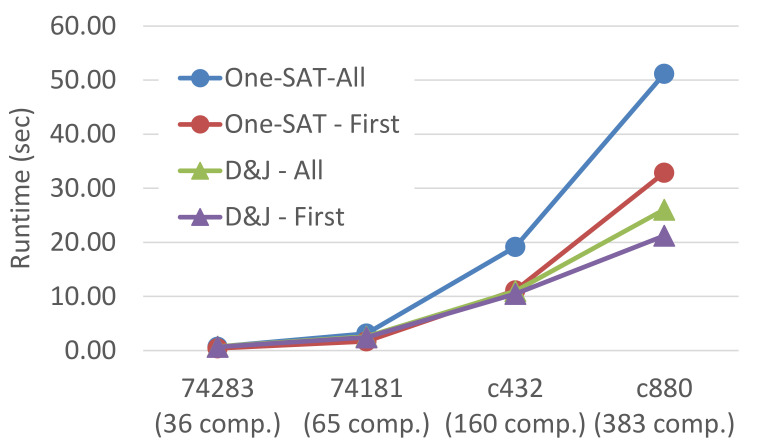
Runtime as a function of the system’s size.

Figure 3 shows runtime results in seconds (*y*-axis) for different systems, ordered by increasing size, averaging over all experiment configurations. D&J is shorthand for divide-and-join-MC. Series whose names end with “-First” and “-All” correspond to results for finding the first MC and all MC diagnoses, respectively.

The results show several interesting trends. First, the runtime of one-SAT becomes significantly larger than that of divide-and-join in larger systems, highlighting the benefit of using divide-and-join for the harder problems. Second, the time gap between finding the first diagnosis and all diagnoses is significant in one-SAT but less so in divide-and-join. This is because one-SAT first searches for a single MC diagnosis and then asks the SAT solver to find all other MC diagnoses. In contrast, divide-and-join computes *all* MC diagnoses for every observation while searching for the first MC diagnoses. Thus, the extra work one-SAT does between finding a first MC diagnosis and finding all of them is smaller than in the divide-and-join algorithm, and consequently the gap between finding a single MC diagnosis and a finding all of them is smaller. Following, we focus on the runtime of finding all MC diagnoses and study the impact of different parameters.

**Figure 4:**
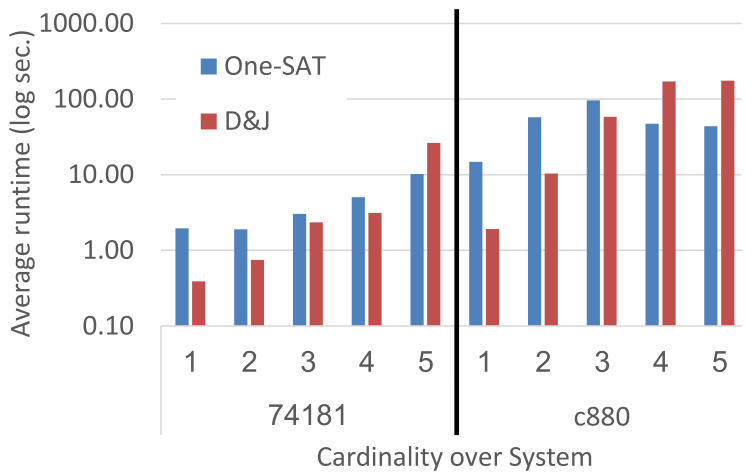
Runtime as a function of the MC diagnosis.

Figure 4 shows runtime results as a function of the MC diagnoses’ cardinality, for systems 74181 and c880 and $p_{int}=0.7$ (other configurations showed similar trends). Interestingly, the runtime divide-and-join grows faster than that of one-SAT as the MC grows. Divide-and-Join is even slower than one-SAT for cardinality 5 and even 4 in c880. This is reasonable since higher cardinality suggests that more observations exhibited abnormal behavior and therefore the join operation will be more time consuming. By contrast, when the cardinality is small this suggests that some of the observations will not even exhibit abnormal behavior. The divide-and-join algorithm is especially suited for such cases, as these observations will not contribute any diagnosis and the join operation can simply skip them.

**Figure 5:**
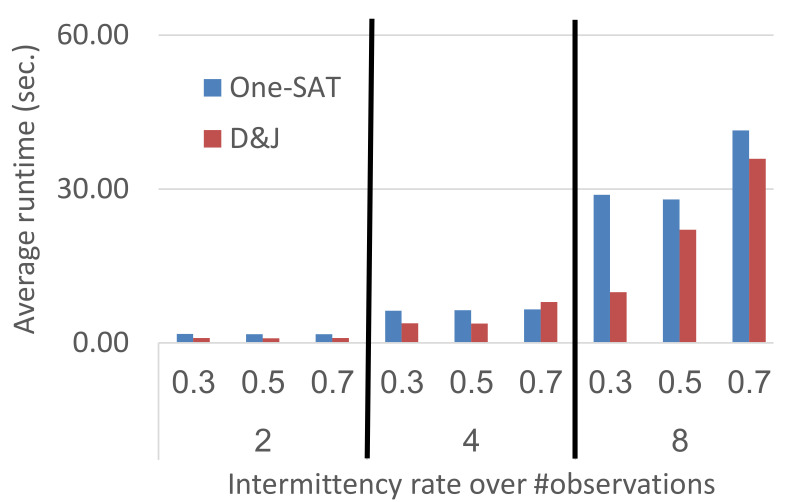
Runtime as a function of the intermittency level ($p_{int}$) and #obs.

Lastly, Figure 5 shows the runtime of both algorithms for problems on the c880 system with different number of observations—2, 4, and 8—and different values of $p_{int}$—0.3, 0.5, and 0.7. As expected, the runtimes of both algorithms increase with the number of observations. The intermittency rate—$p_{int}$—had different effect on the different algorithms. For one-SAT, $p_{int}$ almost had no effect on the overall runtime. By contrast, higher $p_{int}$ values resulted in significantly longer runtimes for divide-and-join-MC. This is understandable, as having lower intermittency rate means fewer components act abnormally, thus resulting in more observations that did not have any diagnosis, making the join operation easier. Similar trends were observed in all the benchmark systems in most configurations.

### Discussion

The following conclusions can be drawn from the experiments:

1. divide-and-join approach is faster than one-SAT for the harder problems (with larger set of components).
2. The time gap between finding the first diagnosis and all diagnoses is significant in one-SAT but less so in divide-and-join.
3. The divide-and-join algorithm is especially suited for diagnosis problems with low cardinality.
4. divide-and-join is faster for cases with fewer abnormal behaviors—smaller MC and $p_{int}$, while one-SAT is more appropriate for cases in which there are many abnormal behaving components.

We believe that these results are significant since they show the benefits of each approach as dependent of the configuration of the system. For instance, for large systems with expected low cardinality and intermittency level, we will prefer divide-and-join than one-SAT. These results are orthogonal to *sequential diagnosis*. Sequential diagnosis is a diagnosis problem in which observations are generated sequentially. Most previous work on sequential diagnosis focused on which observation to generate to reduce the number of diagnoses [9,30,31,32,33], or to learn more about the probabilities of components to be faulty [34]. The results in our work can be used by sequential diagnosis algorithm to allow them to process faster the sequence of observations they receive.

## 7. Conclusions and Future Work

In this paper, we studied the MO-MBD problem, showing that in this problem all four configurations of WFM/SFM and intermittent/non-intermittent are possible. For the specific case of Int.+WFM we proposed two SAT-based algorithms—one-SAT and divide-and-join. There is no dominant algorithm, but we investigated empirically under which conditions each algorithm is superior. Future work will extend one-SAT and divide-and-join to the other fault mode and intermittency configurations (Int.+SFM, NonInt+WFM, and NonInt.+SFM). In particular, adapting NonInt.+WFM is expected to be challenging, as it requires encoding defining that a faulty behavior is consistent without any notion of what the faulty behavior will be.

## Figures and Tables

**Table 1:** A summary of a component’s faulty behavior in the different configurations of WFM/SFM and Int./NonInt.

	WFM	SFM
Int.	Faulty behavior is unconstrained	Must follow a behavior mode but mode can differ between observations
Non-Int.	Not constrained by faulty behavior modes but must be consistent across observations	Must follow a single behavior mode across all observations

**Table 2:** The Benchmark suite: systems 74XXX and ISCAS-85.

Name	|*COMPS*|	in	out
74181	65	14	8
74283	36	9	5
c432	160	36	7
c880	383	60	26

## Data Availability

Not applicable.
